# Substantial loss of T cells upon lymphocyte isolation from heparin-anticoagulated peripheral blood

**DOI:** 10.3389/fimmu.2025.1665686

**Published:** 2025-09-02

**Authors:** Victoria Berg, J. Alexander Ross, Maria Dampmann, Ralf Küppers, Bettina Budeus

**Affiliations:** ^1^ Institute of Cell Biology (Cancer Research), Medical Faculty, University of Duisburg-Essen, Essen, Germany; ^2^ Therapy Research in Neurogeriatrics, Chair of Geriatric Medicine, University of Duisburg-Essen, Essen, Germany; ^3^ Center for Translational Neuro- and Behavioral Sciences, University Hospital Essen, University of Duisburg-Essen, Essen, Germany; ^4^ Department of Hematology and Stem Cell Transplantation, University Hospital Essen, University of Duisburg-Essen, Essen, Germany

**Keywords:** EDTA, heparin, lymphocyte isolation, peripheral blood, T lymphocytes

## Abstract

Analysis of lymphocytes usually involves the usage of blood anticoagulants, with the type of anticoagulant often determined by clinical routines, without testing which anticoagulant is ideal for the research question. We systematically compared the effects of ethylenediaminetetraacetic acid (EDTA) and heparin anticoagulation on recovery and composition of peripheral lymphocyte populations. This comprehensive analysis of the effect of heparin and EDTA on recovery of different lymphocyte subsets has, to our knowledge, not been performed previously. On average, the recovery of lymphocytes is lower in heparin-treated samples. This is due to a loss of T cells, as the absolute numbers of T cells is reduced by 38% in heparin-treated samples compared to EDTA-treated samples, whereas there was no significant loss of B cells observed in the heparin-treated samples. Several T cell subsets, such as memory T cell subsets, are significantly affected. We analysed different steps of the lymphocyte isolation protocol to clarify in which step cells are lost. Losses during several isolation steps showed biases in cell composition, and a significant amount of cells may be lost, e.g. during density gradient centrifugation. However, these losses were generally similar in EDTA- and heparin-treated samples, arguing for an additional mechanism of cell loss. In conclusion, isolation of mononuclear cells from peripheral blood with heparin as anticoagulant causes a partial loss of T cells, with particular subsets preferentially affected, so that there is a risk that the composition of the lymphocyte compartment may be inaccurately assessed when using heparin-treated blood.

## Introduction

A wide range of blood anticoagulants is commercially available, each with its own advantages and drawbacks. For clinical applications, standard procedures depend on the correct choice of anticoagulant. However, knowledge on appropriate choice of anticoagulant is often limited in research settings. This choice is further complicated by a diversity of experimental factors that must be considered, and a desire to make sample procurement easier for all involved parties. Additionally, leftover samples from clinical diagnostics may be used in some cases (1), so that there is no free choice in the anticoagulant for the researchers.

The two commonly used anticoagulants are ethylenediaminetetraacetic acid (EDTA) and ammonium heparin. EDTA is generally used for applications in which blood cell morphology and surface structures need to be preserved. This includes applications such as blood group determinations and complete blood counts (2). Lymphocytes are stable in EDTA-anticoagulated blood for up to 24 h, but viability and cell surface markers may deteriorate afterwards, which may, for example, affect results of immunophenotyping studies. In heparin-treated blood, lymphocytes are stable for longer periods of time (3). EDTA is available in two different formulations, depending on the number of chelated potassium ions: K_3_EDTA and K_2_EDTA. The two types generally perform similarly enough to be interchangeable (4).

Heparins are a group of sulfated glycosaminoglycans of variable lengths (5). Unlike EDTA, heparin is not a strong chelating agent, but instead directly acts on the coagulation cascade by forming complexes with antithrombin and potentiating its fibrinolytic functions, thus inhibiting coagulation (5). As heparin is not a strong chelating agent, ion levels stay constant in the presence of heparin. Therefore, heparin is, for example, used for electrolyte and pH testing as well as blood gas analysis (6). It is also the preferred anticoagulant for assaying immune cell functions, such as calcium flux analysis, as it does not affect extracellular Ca^2+^ concentrations like EDTA does. Ca^2+^ is an important second messenger in immune cells, and appropriate levels are essential for activation and proliferation of lymphocytes. The amount of extracellular Ca^2+^ can influence these processes, with low extracellular Ca^2+^ inhibiting proliferation *in vitro* (7). Ca^2+^ is also required for cell adhesion, and EDTA treatment might therefore abrogate integrin-mediated adhesion (8).

In a clinical setting, using an improper anticoagulant can lead to distorted test results. All anticoagulants have drawbacks, as all of them must disrupt the physiological process of coagulation in some way (9). This is also true for research applications. For example, heparin can interfere with certain assays, such as specific enzymatic assays and immunoassays (10–12) and both excess heparin and EDTA can inhibit polymerase chain reactions, which includes next-generation sequencing applications such as RNASeq (13, 14). Previous studies comparing the effects of different anticoagulants (15–17) mostly focussed on assays in which anticoagulants are known to interfere. However, the effects of anticoagulants on lymphocyte numbers and subset distribution are not as well characterized, and few studies have interrogated the effects of anticoagulants on lymphocyte subtype composition, for example Diks et al. (18), who focused on flow-cytometric concerns such as delayed acquisition times. To our knowledge, no systematic analysis of the effects of different anticoagulants on varied lymphocyte subsets has been published. Working with primary material from patients often severely limits the amount of available material, e.g. due to ethical concerns, patient health, priority of diagnostic testing etc., making it highly important to maximize cell recovery.

Routine blood draws in clinics often call for either ammonium heparin or EDTA as anticoagulants, which are also the most readily available anticoagulants for research. We analysed the usefulness of both anticoagulants in flow cytometry sample preparation. We compared the effects of the anticoagulants on total cell counts and the composition of lymphocyte subsets as determined by flow cytometry.

## Materials and methods

### Blood draws

Blood was drawn from 17 healthy individuals after informed consent was given. Peripheral blood was obtained via venipuncture in the cubital fossa with winged infusion sets (Sarstedt, Nümbrecht, Germany). Sarstedt S-Monovettes (NH_4_-Heparin or K_3_EDTA, Sarstedt) were filled using the aspiration method (19). Three vials each were obtained for either type of collection tube, totalling about 40 ml of blood. Collection tubes were incubated at room temperature for 1 hour, simulating a typical waiting time between blood draw in a clinical setting and analysis and allowing for equilibration to room temperature. Blood volumes were determined using a serological pipette (Sarstedt). The EDTA tubes contain 100 µl of EDTA solution, which was dismissed in the volume calculation.

### Cell isolation

Peripheral blood mononuclear cells (PBMC) were isolated via density gradient centrifugation (DGC). Briefly, 2 ml of Dulbecco’s phosphate buffered saline (PBS) (without Mg^2+^ or Ca^2+^, Pan Biotech, Aidenbach, Germany) with 0.5% w/v bovine serum albumin (BSA), fraction V (PBS/BSA) was added to each blood collection tube and mixed by inverting. Blood was transferred to a tissue culture dish to combine all tubes and ease washing of the heparin-coated beads, then layered over 15 ml of Pancoll centrifugation medium (density 1.077 g/cm^3^, Pan Biotech) in 50 ml reaction tubes (Sarstedt). Blood collection tubes and tissue culture dishes were washed three times each with 2 – 4 ml of PBS/BSA to maximize blood transfer. Centrifugation was performed for 35 minutes at 400 × g (18°C, brake set to 5/9). The PBMC layer was collected and washed with PBS/BSA.

Erythrocyte lysis was performed by adding erythrocyte lysis buffer (150 mM ammonium chloride, 10 mM potassium bicarbonate, 0.1 mM EDTA in *a. bidest*) at four times the volume of the blood sample to the sample in 50 ml reaction tubes, shaking the samples thoroughly. Lysis was performed until the samples turned dark and translucent, which typically occurs after 10 to 12 minutes. Samples were then centrifuged at 400 × g for 5 minutes, and the supernatant was discarded. The cell pellets were resuspended and washed twice with 50 ml PBS/BSA for 10 minutes at 400 × g.

### Flow-cytometric and statistical analysis

Cell counts were obtained with a CytoFLEX S flow cytometer in a four-laser NVBR configuration (Beckman-Coulter, Brea, CA, USA), gating on lymphocyte singlets. Cells were stained with two different antibody panels (**Supplementary Table S1**) that allowed differentiation between major B cell and T cell subsets, and measured with the CytoFLEX S. A viability dye was not included, and the FSC/SSC parameters were used to exclude debris and dead cells. Compensation and gates were adjusted separately for each sample (**Supplementary Figures S1**, **S2**). At least 50,000 events were recorded for each sample. Paired Wilcoxon signed rank tests were performed to compare the measured lymphocyte populations of heparin and EDTA samples. Calculations were performed with R (version 4.3.1), using base R and the packages ggpubr, ggplot2, and ggstatsplot.

## Results

### Cohort

A cohort of 17 healthy individuals was enrolled in this study, with a median age of 37.4 years (range: 23 to 61 years), skewed towards male participants (70.6% male). Participants with current, even mild, infections, e.g. upper respiratory tract infections, were excluded. The full dataset is provided in **Supplementary Table S2**.

### Lower recovery of lymphocytes from heparin- than from EDTA-anticoagulated blood

After DGC, a thin layer of PMBCs forms at the interphase of plasma and Pancoll. The appearance of this layer varied between anticoagulants, with the PBMC layer being more diffuse in heparin samples than in EDTA samples. Slight differences in plasma colour were also noted.

**Figure 1:**
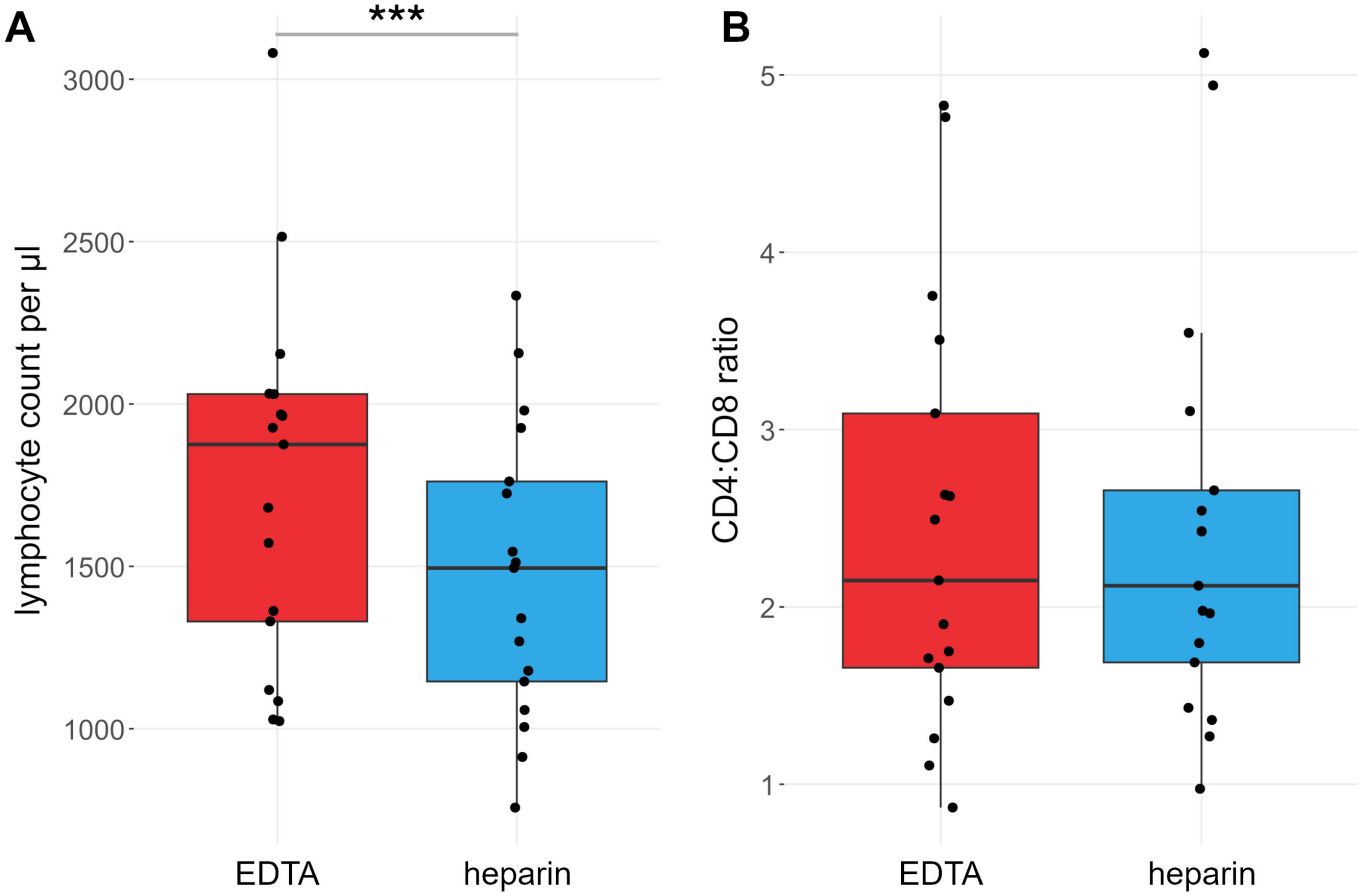
Lymphocyte counts are different between heparin- and EDTA-anticoagulated blood. Red: EDTA, blue: heparin. **(A)** Lymphocyte counts per µl blood. **(B)** Ratio of CD4^+^ vs CD8^+^ T cell counts. *P* values calculated by paired Wilcoxon rank sum test. ***p < 0.001; *ns*, not significant. *n = 17*.

For each sample, a lymphocyte count per µl blood was calculated. On average, lymphocyte counts were 21% higher (range: 17% lower to 54% higher) in EDTA-anticoagulated blood than in heparin-anticoagulated blood (mean_Heparin_ = 1386.7 cells/µl, mean_EDTA_ = 1713.5 cells/µl). This difference was highly significant at *p* < 0.001 (**Figure 1A**).

### B cell subpopulation ratios are not differentially affected by EDTA or heparin

**Figure 2:**
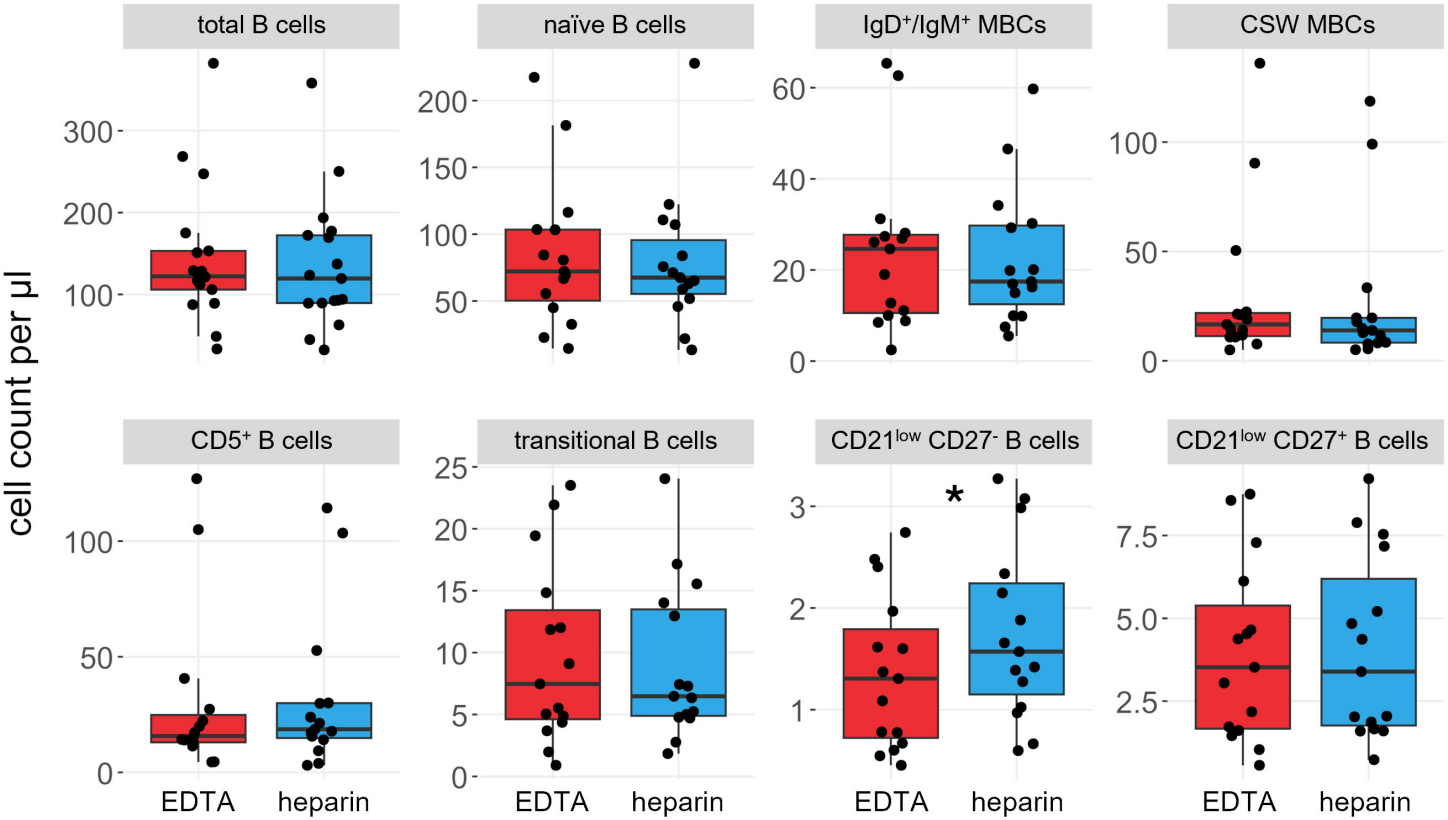
B cell subsets are not significantly influenced by choice of anticoagulant. Boxplots and jittered dotplots of flow cytometry data. B cell panel shows the same data as the B cell panel in **Supplementary Figure S3**. Red; EDTA, blue; heparin. P values calculated by paired Wilcoxon rank sum test. *p < 0.05. *n = 15*. MBCs, memory B cells; CSW, class-switched.

We compared the effects of EDTA and heparin on the main B cell subsets in 15 of the 17 individuals, namely naïve B cells (IgD^+^ CD27^-^), IgM^+^/IgD^+^ (IgD^+^ CD27^+^) and class-switched memory B cells (CD27^+^ IgG/IgA^+^), CD5^+^ mature B cells (CD5^+^ CD38^low^), transitional B cells (CD38^high^), and CD21^low^ CD27^-^ and CD21^low^ CD27^+^ B cells (**Figure 2**, see **Supplementary Figure S1** for gating strategy). The total number of B cells did not differ significantly between the sample types (mean_Heparin_ = 149.0 cells/µl, mean_EDTA_ = 155.4 cells/µl, *p* = 0.22), and most B cell subsets did not show differences in total number. Only the CD21^low^ CD27^-^ subset showed significantly higher cell numbers in heparin-treated samples, though the effect size was very small (mean_Heparin_ = 1.6 cells/µl, mean_EDTA_ = 1.3 cells/µl, *p* = 0.026, **Figure 2**).

### Substantial losses of T cells in heparin-anticoagulated blood samples, with distinct effects on distinct T cell subsets

**Figure 3:**
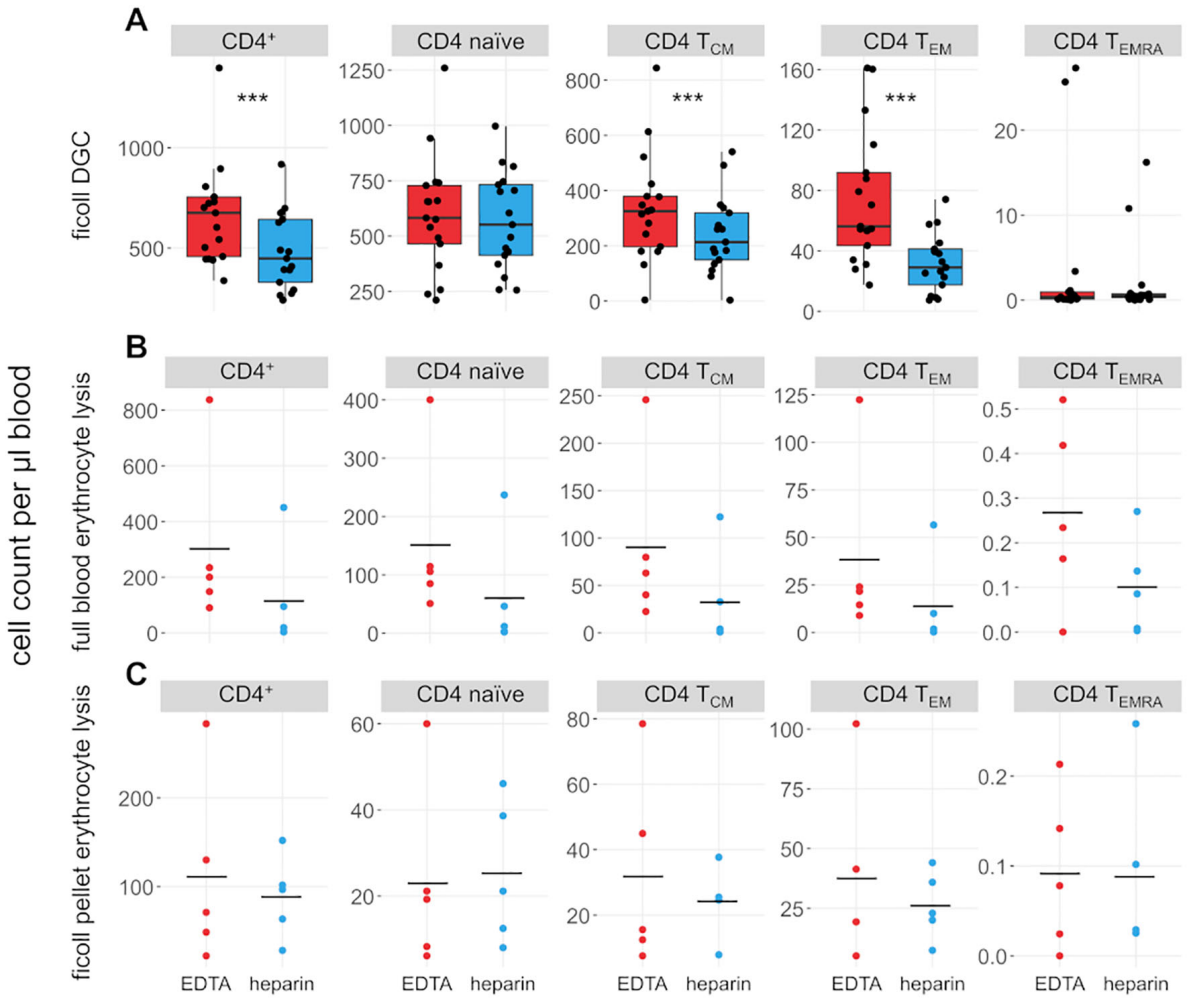
CD4^+^ T cell subsets are influenced by choice of anticoagulant. **(A)** Boxplots and jittered dotplots showing flow-cytometric data from lymphocytes isolated by density gradient centrifugation (DGC). *n* = 17. **(B)** Absolute cell counts of lymphocyte subsets isolated by full blood erythrocyte lysis. *n* = 5. **(C)** Absolute cell counts of lymphocyte subsets isolated by erythrocyte lysis of DGC pellet. *n* = 5. EDTA, red; heparin, blue. Horizontal black lines indicate means. P values calculated by paired Wilcoxon rank sum test (due to low *n*, no *p* values were calculated for **(B, C)**. ***p < 0.001.

**Figure 4:**
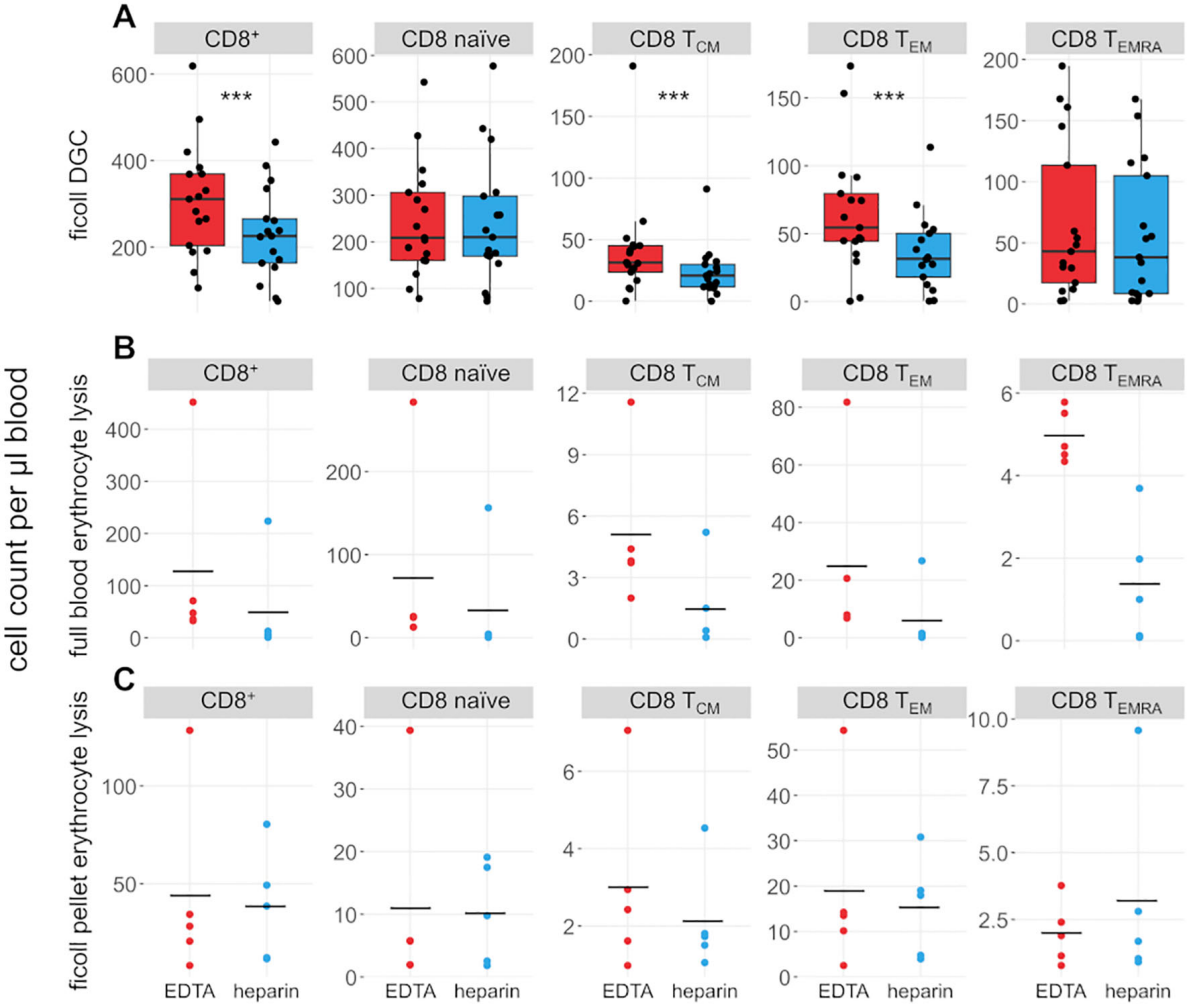
CD8^+^ T cell subsets are influenced by choice of anticoagulant. **(A)** Boxplots and jittered dotplots showing flow-cytometric data from lymphocytes isolated by density gradient centrifugation (DGC). *n* = 17. **(B)** Absolute cell counts of lymphocyte subsets isolated by full blood erythrocyte lysis. *n* = 5. **(C)** Absolute cell counts of lymphocyte subsets isolated by erythrocyte lysis of DGC pellet. *n* = 5. EDTA, red; heparin, blue. Horizontal black lines indicate means. P values calculated by paired Wilcoxon rank sum test (due to low *n*, no *p* values were calculated for **(B, C)**. ***p < 0.001.

We also compared the effects of heparin and EDTA on several T cell subsets (**Figures 3**, **4**). Notably, unlike B cells, the total number of T cells per µl blood was significantly reduced by 38% in heparin samples compared to EDTA samples (mean_Heparin_ = 803.2 cells/µl, mean_EDTA_ = 1070.0 cells/µl, *p* < 0.001). However, the ratio of CD4^+^ T cells versus CD8^+^ T cells is not affected by the chosen anticoagulant (mean_EDTA_ = 2.43 vs mean_Hep_ = 2.44, *p* = 0.96; **Figure 1B**). To elucidate which cells are reduced in heparin samples, we stained additional T cell subsets, including naïve T cells (CD3^+^ CD62L^+^ CD45RA^+^), central memory T cells (T_CM_; CD3^+^ CD62L^-^ CD45RA^+^), effector memory T cells (T_EM_; CD3^+^ CD62L^-^ CD45RA^-^), and effector memory T cells re-expressing CD45RA (T_EMRA_; CD3^+^ CD62L^-^ CD45RA^+^; see **Supplementary Figure S2** for gating strategy). We selected these subsets as a representative overview of the T cell landscape, focusing on the differentiation between CD4^+^/CD8^+^ T cells as well as naïve cells and distinct memory populations. Note that additional CD4^+^ subsets characterized by differential cytokine expression (Th1, Th2, Th17) were also stained and partially overlap with the naïve-memory classification (data not shown). Most T cell subsets were significantly reduced in heparin samples, with the exceptions of CD4^+^ and CD8^+^ T_EMRA_ (**Figures 3**, **4**), T_regs_ and Th2 cells (not shown).

### Some T cell subsets are preferentially retained or lost during different steps of sample preparation

We further explored how T cell subsets were significantly affected by differences in anticoagulation. Note that the following experiments were performed on fewer samples than the main experiment described above (*n* = 5). As the number of T cells was significantly reduced in heparin-anticoagulated samples compared to EDTA-treated blood, we hypothesized that lymphocytes were retained at some point in the process. As the ammonium heparin tubes we used contain plastic beads with a rough surface to expand the available surface area for heparin treatment, it seemed likely that cells were adhering to these beads. Therefore, we thoroughly washed the beads and collection tubes (5 × 7.5 ml PBS/BSA) after a standard DGC was prepared, and performed a second DGC with the washes. Comparing these samples revealed that the T cell composition shifts with additional washes. Subsets such as naive CD4^+^ and CD8^+^ T cells and T_regs_ were reduced, while other such as CD4^+^ and CD8^+^ T_EM_ are overrepresented in late wash steps (**Supplementary Figure S4A**). However, this bias cannot explain the large discrepancy (up to 42%) in cell numbers between EDTA and heparin samples, as only 1 - 2% of total cells are recovered during later washes (**Supplementary Figure S4B**), arguing that cells are mostly lost via other mechanisms.

Erythrocyte lysis was performed on full peripheral blood and the erythrocyte-rich pellet left after DGC (**Figures 3**, **4B, C**) to investigate whether cells are lost during DGC. Higher cell counts in EDTA-anticoagulated blood are obvious in full-lysed blood samples as well, arguing that the loss of cells in heparin-treated samples cannot be primarily dependent on the isolation method. A significant number of lymphocytes was recovered from the DGC pellet, representing 82% and 72.4% of the cells recovered from the DGC interphase in heparin and EDTA samples, respectively. Some lymphocyte subsets are preferentially lost in this step, e.g. CD4^+^ and CD8^+^ T_EMRA_, as evidenced by their overrepresentation in the lysed pellet of the density centrifugation compared to full blood and isolated lymphocytes (relative fraction sizes; data not shown). This effect is similar in both EDTA- and heparin-anticoagulated samples. Therefore, the loss in heparin-anticoagulated samples specifically cannot be explained by preferential loss of T cells in the pellet of the density centrifugation. The upper phase of the samples after DGC, which contains mostly serum and thrombocytes, was also analysed. However, as expected, only few cells (typically 50,000 to 100,000 cells per sample) were present in the phase and thus not further analyzed. In addition, we analyzed the effect of a prolonged centrifugation (1 h vs. the original 35 minutes) in three probands and found that cell yields were increased after 1 h (see **Supplementary Table S2**). This increase was higher in heparin- than in EDTA-anticoagulated samples (11.8% vs. 5.9%). However, the bias between the different anticoagulants was still present, with cell yields in EDTA-treated samples being on average 21% higher than in heparin-treated samples.

Finally, we analysed the supernatant from washing the cells after DGC. Supernatants were collected and centrifuged again for 10 minutes, resulting in a small cell pellet. Some few million cells were typically recovered from the supernatant (mean: 3.26 × 10^6^ lymphocytes, range: 499,000 - 4.95 × 10^6^ lymphocytes). A larger percentage of non-lymphocyte cells was recovered from the supernatants compared to the corresponding DGC samples (48.7% vs. 40.0%) while the number of recovered T cells was increased in the DGC samples (40.1% vs. 32.6% in supernatants). Some T cell subsets were slightly over- (e.g., CD4^+^ naïve, CD4^+^ in general) or underrepresented (CD4^+^ T_CM_, CD8^+^ T_EM_, CD8^+^ in general; **Supplementary Figure S5**) in the supernatant compared to the cells isolated via DGC. However, the effect was similar in both heparin- and EDTA-treated samples.

## Discussion

### Choice of anticoagulant influences both absolute cell counts and lymphocyte composition

In the present work, we determined that EDTA- and heparin-anticoagulated peripheral blood samples differ significantly in their lymphocyte cell counts and composition. On average, T cell counts are 38% lower in heparin- than in EDTA-treated samples. We interrogated all steps of a DGC protocol to ascertain in which step the lymphocytes in heparin-treated samples are lost. The loss of T cells was largely independent of the sample preparation process, as during all steps of the protocol, cell counts were consistently higher in EDTA- than in heparin-anticoagulated samples. However, sample preparation-specific processes may explain why some T cell subsets are more affected. For example, T_EMRA_ cells were recovered at higher frequencies from lysed density centrifugation pellets. All in all, a picture emerges that certain T cell subsets, such as CD4^+^ and CD8^+^ T_EMRA_ and T_EM_, are more easily pelleted during centrifugation or are preferentially retained on plasticware. We hypothesize that this is due to enhanced adherence, e.g. to other cells, affecting centrifugation, and to plasticware. Ca^2+^ is necessary for integrin-mediated cell adhesion, and its chelation by EDTA might lower adhesion in comparison to heparin-treated samples. However, this effect seems to occur in both heparin- and EDTA-treated samples.

It is possible that lymphocytes die more easily in heparin-treated peripheral blood and that those cells are lost due to this as-of-yet unidentified mechanism. However, it is important to keep in mind that the cells recovered from EDTA-treated blood likely also do not represent the full amount of lymphocytes physiologically present in peripheral blood, as technical losses may still occur which are difficult to quantify due to lack of an unprocessed control.

Since the CD4:CD8 ratio is similar in either sample type, it is probably not a specific TCR coreceptor-mediated effector function of T cell subsets that primes them for destruction or conversion. However, the affected subsets seem to be functionally defined. This might possibly be due to activation of lymphocytes by heparin. Heparin is a ligand of ALK (20) and FGFR4 (21), interacting with FGF itself as well (22) and might activate lymphocytes via this pathway. This hypothesis may be tested in future studies, e.g. by RNA sequencing of primary lymphocytes or lymphocyte-derived cell lines exposed to heparin. Heparin is also discussed as a signalling molecule secreted by mast cells for communication with other immune cells (21).

Upon immunoreceptor activation, calcium influx into lymphocytes replenishes internal Ca^2+^ storage (23). Lymphocytes are activated upon blood sampling, e.g. due to puncture of a blood vessel and the associated physiological response and contact with plasticware. Since EDTA is a chelator of Ca^2+^, it effectively removes Ca^2+^ from the extracellular medium. Ongoing activation may thus be abrogated in EDTA-treated samples and increased in heparin-treated blood samples. Conversely, Kwarteng and colleagues (24) showed a small, but significant increase in T cell activation in EDTA-, but not heparin-treated blood samples within 4 hours of sampling. However, after 1 hour, the fraction of activated T cells was generally higher in heparin-treated samples. Including activation markers in a subsequent study may shed more light on the precise nature of our observations.

### Choosing the right anticoagulant is essential for experiment optimization

The present work highlights the importance of choosing an appropriate anticoagulant for the planned assays and experiments. Using EDTA will maximize the number of lymphocytes available, especially preventing the loss of cells of particular T cell subsets, making this anticoagulant suitable for flow-cytometric analyses of lymphocyte subsets and settings with very limited sample volumes. A major drawback is the short period of storage possible in EDTA-treated samples, as surface markers may be unstable and the total cell numbers may decrease if stored at room temperature for more than 24 h (18). Heparin, on the other hand, is suitable for functional assays that may be impeded by the lack of Ca^2+^, such as calcium flux assays ascertaining depletion of intracellular Ca^2+^ storage as an indicator of lymphocyte activation. In addition, heparin allows longer storage periods, which may be important when collecting fresh blood samples in remote locations. In general, the sampling method should be considered carefully to optimize the experimental parameters. As the results of this study may not be applicable to all other lymphocyte isolation protocols, it may be helpful to test different anticoagulants with the protocols of choice. Testing against clinical parameters determined by standardized tests, such as CD4 T cell count, leukocyte/lymphocyte counts etc., may be of use to validate the chosen methods.

## Conclusion

Our study shows that the choice of anticoagulant is essential for cell recovery. EDTA- and heparin-anticoagulated blood can dramatically differ in cell recovery, and we observed a specific loss of T cells in heparin-anticoagulated samples. Saliently, not only total cell numbers, but also the composition of different T lymphocyte subsets is significantly altered. This study exemplifies the need for a more comprehensive approach to sample preparation and processing in immune phenotyping studies. Further research should focus on recovery of other cell types, such as cells of the myeloid lineage, and on including different anticoagulant formulations and manufacturers to develop standardized recommendations for anticoagulants and processing protocols from which the immunological research community may benefit, i.e. by helping to ensure consistency and reproducibility across studies.

## Data Availability

The original contributions presented in the study are included in the article/**Supplementary Material**. Further inquiries can be directed to the corresponding authors.
