# Supplementary material for: Substantial loss of T cells upon lymphocyte isolation from heparin-anticoagulated peripheral blood
Source: Front Immunol. 2025 Sep 2;16:1665686. doi: 10.3389/fimmu.2025.1665686 (PMC12436355; doi:10.3389/fimmu.2025.1665686)
Supplement: Supplementary file 1 [file DataSheet1.pdf]

Supplement for

Substantial loss of T cells upon lymphocyte isolation from heparin-anticoagulated peripheral blood

Victoria Berg, J. Alexander Ross, Maria Dampmann, Ralf Küppers, Bettina Budeus

Frontiers in Immunology

This supplement contains the following items:

Supplementary Figure S1: Gating strategy for B lymphocytes

Supplementary Figure S2: Gating strategy for T lymphocytes

Supplementary Figure S3: B and T cells are differentially affected by anticoagulants

Supplementary Figure S4: Lymphocyte composition of cells from different isolation steps

Supplementary Figure S5: Lymphocyte composition of cells from Pancoll DGC isolation and supernatant from washing the isolated cells

Supplementary Table S1: Antibody panel for flow cytometry

Supplementary Table S2: Full dataset

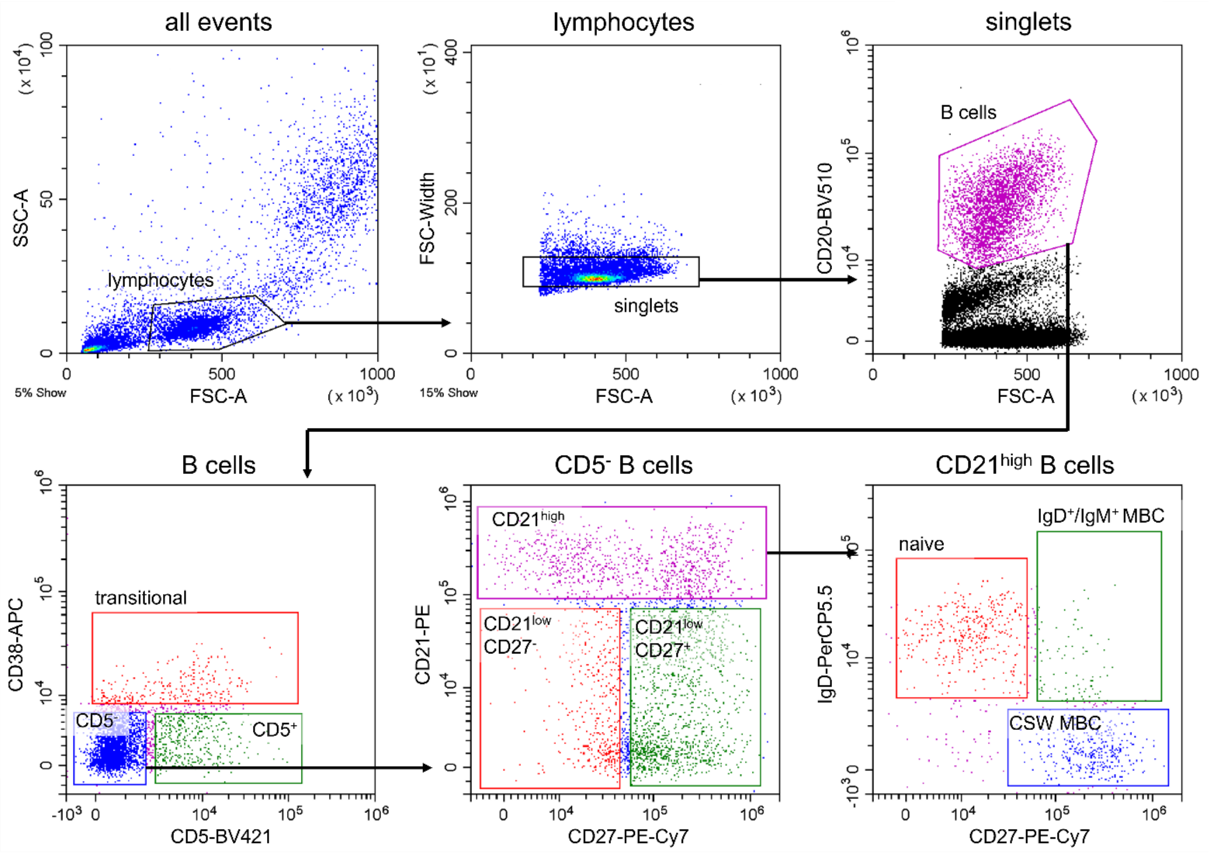

Supplementary Figure S1: Gating strategy for B lymphocytes. Living lymphocyte singlets are selected via the forward scatter-area (FSC-A) and side scatter-area (SSC-A) parameters. Doublets are excluded by the FSC-width (FSC-W) parameter. $CD20^{+}$ B cells are divided into $CD5^{-}$, $CD5^{+}$ mature and $CD38^{high}$ transitional B cells. The $CD5^{-}$ B cells are further divided into the two $CD21^{low}$ subsets and the $CD21^{high}$ subset. The latter is then further differentiated into naïve B cells, IgD^{+}/IgM^{+} memory B cells (MBCs) and class-switched (CSW) MBCs.

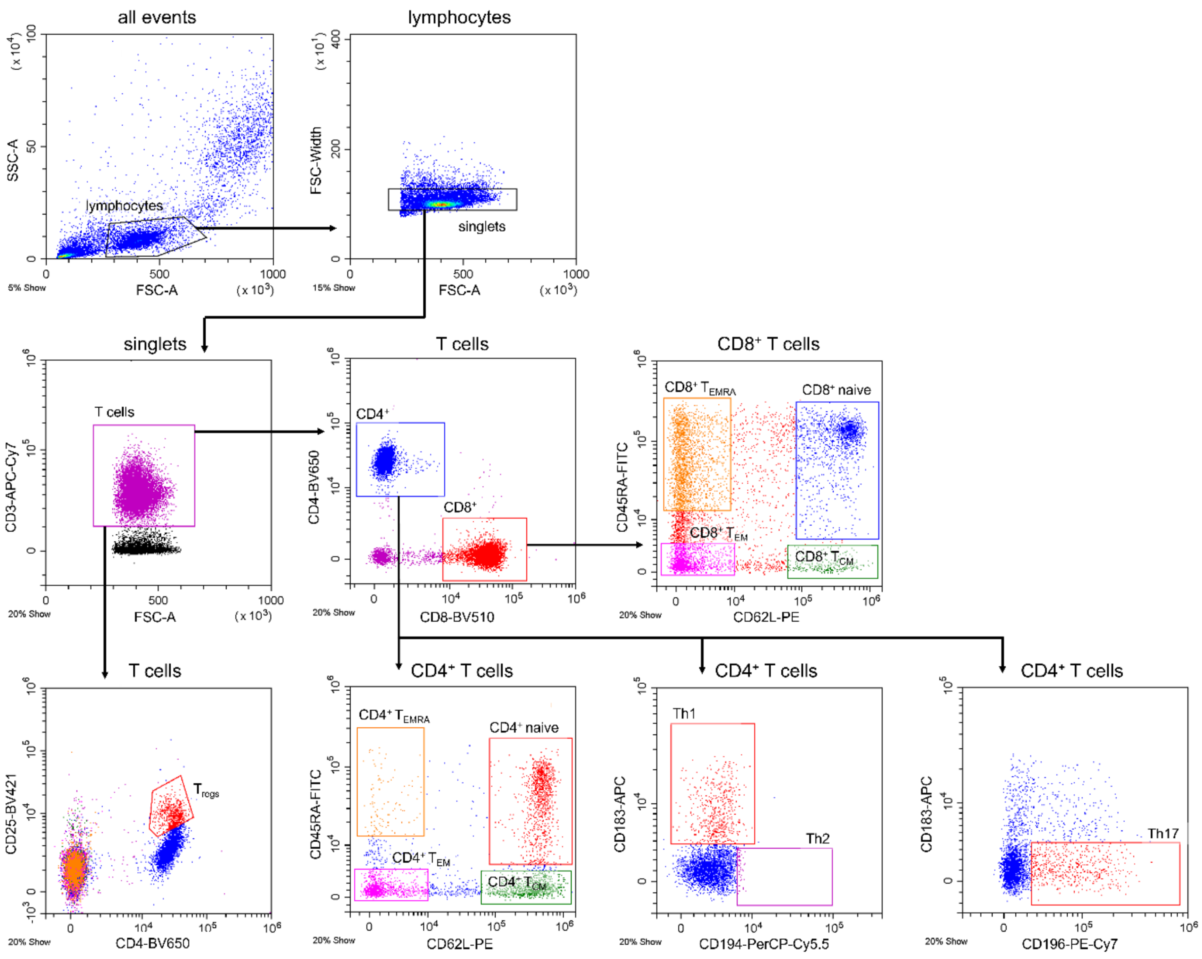

Supplementary Figure S2: Gating strategy for T lymphocytes. Lymphocyte singlets are selected as described in Suppl. Fig. S1. CD3$^+$ T cells are divided into CD4$^+$ and CD8$^+$ T cells. CD25$^+$ CD4$^+$ T cells are identified as Tregs. Both CD4$^+$ and CD8$^+$ T cells are differentiated into naïve, T_{CM}, T_{EM} and T_{EMRA} subsets by the CD62L/CD45RA staining. In addition, the CD4$^+$ T cells are classified into Th1, Th2 and Th17 helper T cells.

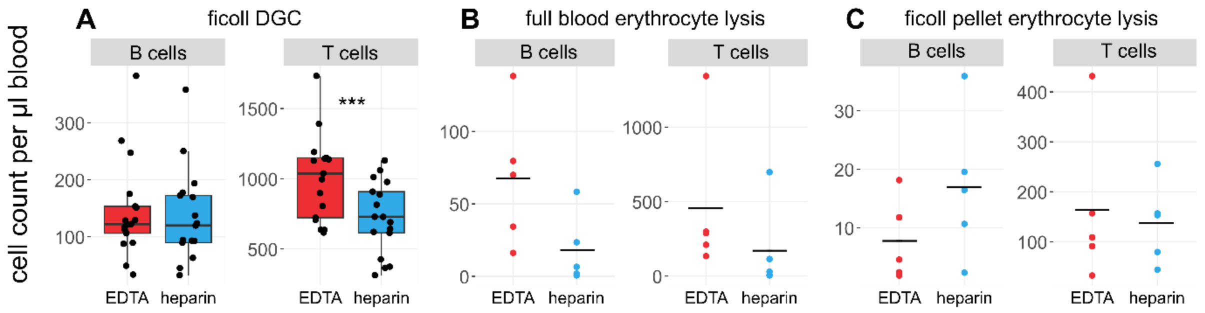

Supplementary Figure S3: B and T cells are differentially affected by anticoagulants. A) Boxplots and jittered dotplots showing flow-cytometric data from lymphocytes isolated by density gradient centrifugation (DGC). B cell panel shows the same data as the total B cell panel in Fig. 2. n_{Bcells} = 15, n_{Tcells} = 17. B) Absolute cell counts of lymphocyte subsets isolated by full blood erythrocyte lysis. n = 5. C) Absolute cell counts of lymphocyte subsets isolated by erythrocyte lysis of DGC pellet. n = 5. EDTA, red; heparin, blue. Horizontal black lines indicate means. *P* values calculated by paired Wilcoxon rank sum test (due to low n, no p values were calculated for B and C). ***$p < 0.001$.

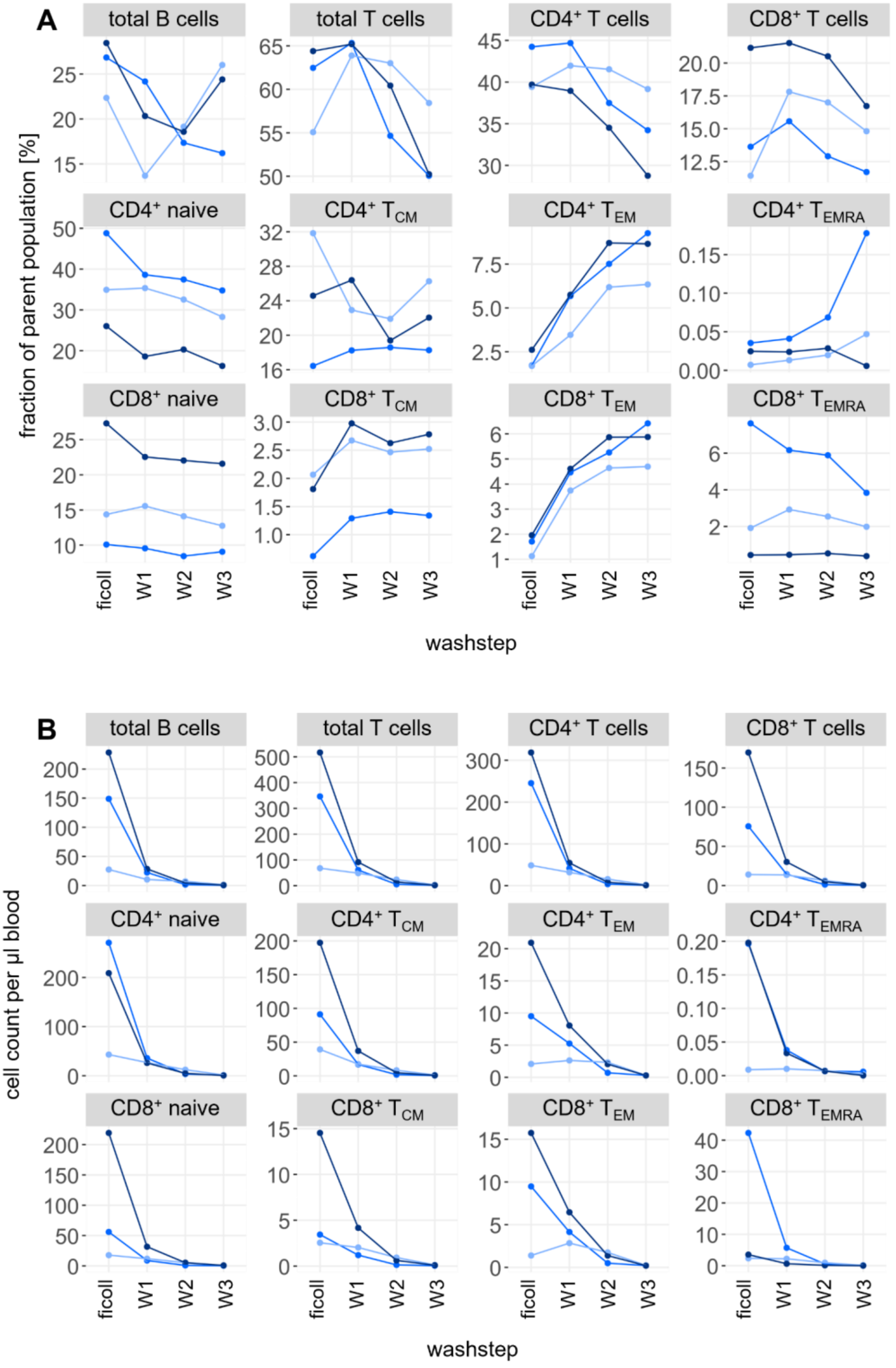

Supplementary Figure S4: Lymphocyte composition of cells from different isolation steps. A) Lymphocyte counts expressed as fractions of the parent population show that subset composition changes with additional washes. B) Total cell counts show that the cell numbers are drastically reduced with every additional wash. Heparin-treated blood. Each graph denotes one sample, *n* = 3.

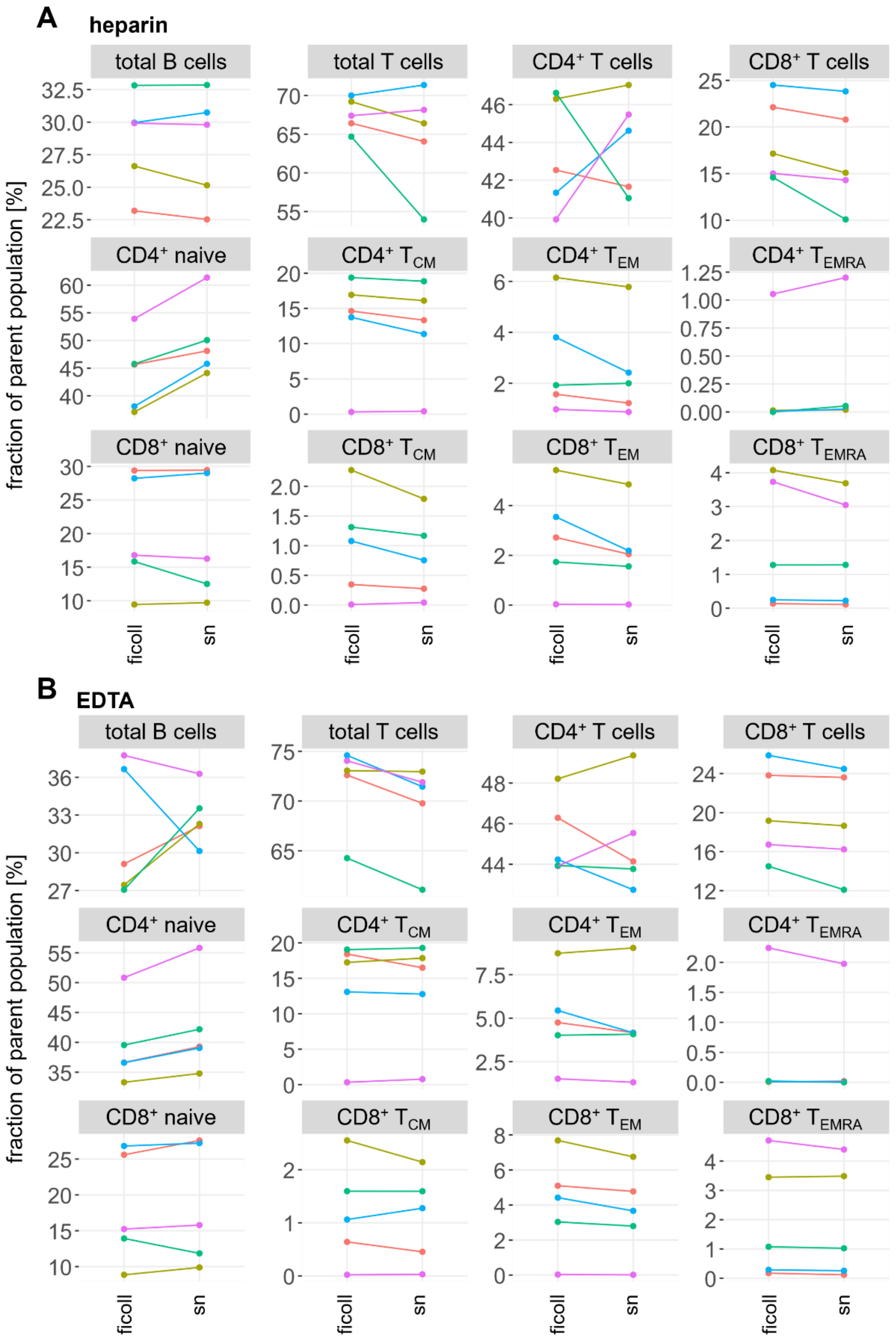

Supplementary Figure S5: Lymphocyte composition of cells from Pancoll DGC isolation and supernatant from washing the isolated cells. A) Heparin-treated samples, B) EDTA-treated samples. Slopes are generally similar between both anticoagulants. sn: supernatant. Each graph denotes one sample, *n* = 5.

Supplementary Table S1: Antibody panel for flow cytometry

Antibody	Conjugate	Clone	Species & Isotype	Company	Panel
anti-hu CD3	APC-Cy7	SK7	mouse IgG1, κ	Biolegend	T cells
anti-hu CD4	BV650	SK3	mouse IgG1, κ	BD	T cells
anti-hu CD8	BV510	SK1	mouse IgG1, κ	BD	T cells
anti-hu CD25	BV421	M-A251	mouse IgG1, κ	BD	T cells
anti-huCD45RA	FITC	5H9	mouse IgG1, κ	BD	T cells
anti-hu CD62L	PE	DREG-56	mouse IgG1, κ	BD	T cells
anti-hu CD183	APC	1C6	mouse IgG1, κ	BD	T cells
anti-hu CD194	BB700	1G1	mouse IgG1, κ	BD	T cells
anti-hu CD196	PE-Cy7	11A9	mouse IgG1, κ	BD	T cells
anti-hu CD5	BV421	L17F12	mouse IgG2a, κ	Biolegend	B cells
anti-hu CD20	BV510	2H7	mouse IgG2b, κ	Biolegend	B cells
anti-hu CD21	PE	B-ly4	mouse IgG1, κ	BD	B cells
anti-hu CD27	PE-Cy7	O323	mouse IgG1, κ	BD	B cells
anti-hu CD38	APC	HIT2	mouse IgG1, κ	BD	B cells
anti-hu IgD	PerCP-Cy5.5	IA6-2	mouse IgG2a, κ	Biolegend	B cells
anti-hu IgM	BV650	MHM-88	mouse IgG1, κ	Biolegend	B cells
anti-hu IgG	FITC	IS11-3B2.2.3	mouse IgG1, κ	Miltenyi	B cells
anti-hu IgA	FITC	IS11-8E10	mouse IgG1, κ	Miltenyi	B cells
